# Ten‐year trends of national healthcare costs of asthma, allergic rhinitis, and atopic eczema in 3 million Norwegians

**DOI:** 10.1111/all.15225

**Published:** 2022-02-01

**Authors:** Christine Louise Parr, Wenche Nystad, Øystein Karlstad, Knut Øymar, Arnulf Langhammer, Per Nafstad, Torbjørn Wisløff

**Affiliations:** ^1^ Norwegian Scientific Committee for Food and Environment Norwegian Institute of Public Health Oslo Norway; ^2^ Department of Chronic Diseases and Ageing Norwegian Institute of Public Health Oslo Norway; ^3^ Department of Pediatrics Stavanger University Hospital Stavanger Norway; ^4^ Department of Clinical Science University of Bergen Norway; ^5^ Department of Public Health and Nursing Norwegian University of Science and Technology Trondheim Norway; ^6^ Department of Community Medicine and Public Health University of Oslo Oslo Norway; ^7^ Health Services Research Unit Akershus University Hospital Lørenskog Norway; ^8^ Department of Method Development and Analytics Norwegian Institute of Public Health Oslo Norway

## FUNDING INFORMATION

This study was supported by the Norwegian Directorate of Health.

## CONFLICTS OF INTEREST

Øystein Karlstad reports participation in research projects funded by Novo Nordisk and LEO Pharma, all regulator‐mandated phase IV‐studies, all with funds paid to his institution (no personal fees) and with no relation to the work reported in this paper. Arnulf Langhammer has been PI of the Lung Study in HUNT that was partially funded by non‐demanding grants from AstraZeneca. He has participated in some advisory boards for AstraZeneca and GlaxoSmithKline and has given lectures for medical doctors paid by AZ, GSK and Boehringer Ingelheim. He has not received any payment related to the work reported in this paper. Torbjørn Wisløff has done consulting for completely unrelated project on varicella and herpes zoster vaccine for MSD. Christine Louise Parr, Wenche Nystad, Knut Øymar, and Per Nafstad reports no conflicts of interest.

To the editor,

Asthma and allergies pose a significant health and economic burden on many populations.^1^, ^2^, ^3^ Norway has lacked national figures and relied on estimates from other Nordic countries^4^, ^5^ although there are differences in morbidity, health care, and national policies. Therefore, we have estimated time trends in annual diagnosis‐specific costs of asthma, allergic rhinitis, and atopic eczema in the entire Norwegian population aged 0–44 years (3 million individuals) for three areas of the health sector: (1) prescription drugs, (2) specialist health care, and (3) general practitioner (GP) consultations. Diagnoses were based on reimbursement codes in three national health registries (available years), for prescription drugs, the Norwegian Prescription Database (2010–2018), for the specialist health care, the Norwegian Patient Registry (2008–2017), and for general practitioner consultations, the Control and Payment of Health Reimbursement Registry (2006–2016). Thus, 2016 was the latest year with data from all sources. Costs were standardized to 2018.

**FIGURE 1:**
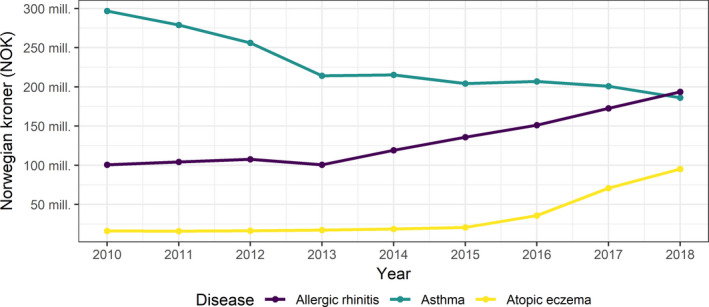
Time trend in annual drug expenditures on asthma, allergic rhinitis, and atopic eczema in the Norwegian population aged 0–44 years, by disease. Norwegian Prescription Database 2010–2018

Drug expenditures decreased for asthma, but increased for rhinitis and eczema, and increased overall from NOK 414 to 475 million in the period from 2010 to 2018 (Figure 1). Costs of specialist health care, on the other hand, were more than halved from NOK 401 to 187 million in a similar time period (2008–2017), mainly due to lower costs of asthma hospitalizations in children, in particular for ages 1–4 years. Costs of GP visits increased from NOK 209 to 239 million (2006–2016), driven by rhinitis and eczema. Total costs of health care (sectors and conditions combined) decreased by NOK 139 million (from 1022 million to 883 million) from 2010 to 2016 (Appendix S1: Table A1, data in all registries). Asthma contributed 61%, rhinitis 26%, and eczema 13% of total costs. By sector, drug expenditures contributed 45%, specialist health care 28%, and GP consultations 27% of total costs.

**FIGURE 2:**
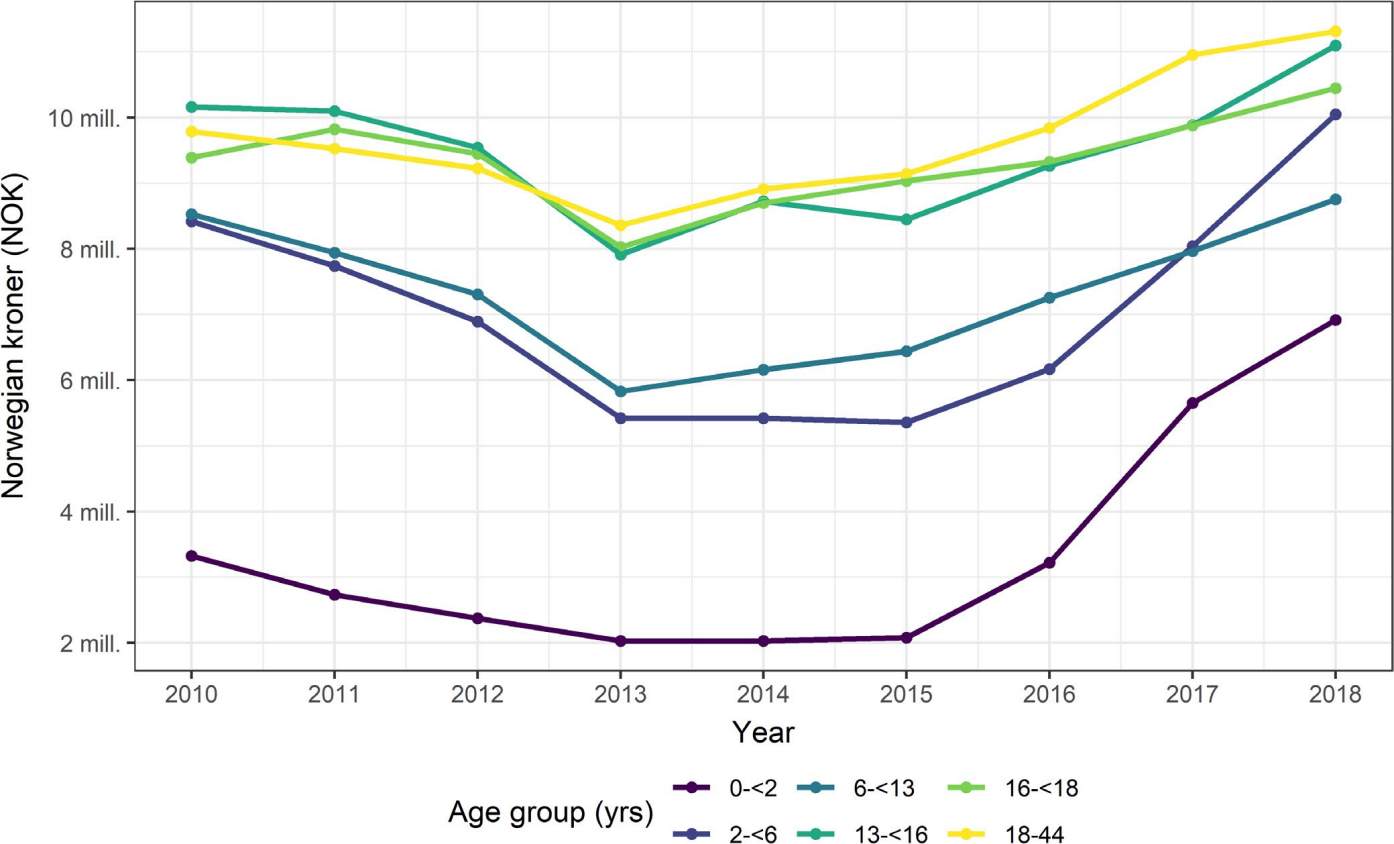
Time trend in the average annual drug expenditures on asthma, allergic rhinitis, and atopic eczema in the Norwegian population aged 0–44 years, by age group. Norwegian Prescription Database 2008–2018

Adolescents and adults have had the highest average drug expenditures per age cohort per year (Figure 2). Since 2015, however, there has been a steep increase for the two youngest age groups. Among infants (0–<2 years), total drug expenditures have more than tripled, from NOK 2.1 million in 2015 to NOK 7.0 million in 2018. The seemingly U‐shaped figure is due to decreases in asthma and increases in both allergic rhinitis and eczema, as shown in Appendix S1: Figure A1.

The average cost of specialist health care per age cohort has decreased in all age groups between 2008 and 2017 (Appendix S1: Figure A2), but most noticeably among children <5 years where costs also have been highest. The jagged pattern among infants (age <1 year) is consistent with bronchiolitis caused by viral infections, where outbreaks may cause large annual variations (data not shown).

Total costs for GP visits increased with 14%, from 210 million in 2006 to 240 million in 2016 (Appendix S1: Figure A3). Asthma costs decreased by 9.1% but remained the highest. Costs of atopic eczema and allergy increased steadily through the period (Appendix S1: Figure A3). The average cost of GP consultations per age cohort was highest in children aged 0–<4 and 4–<8 years and lowest in adults, reflecting that young children have more contacts with GPs than adults (Appendix S1: Figure A4). Costs increased with 31% over the period among adults but were relatively stable in other age groups.

In all sectors, costs have increased for allergic rhinitis and atopic eczema but decreased for asthma (see Appendix S1 for discussion of results). The current analysis will provide an important baseline assessment for future evaluations of public health measures in Norway, particularly when assessing the impact of recently established new centers for asthma, allergy, and hypersensitivity reactions within each of the regional hospital trusts.^6^

## Supporting information

Appendix S1
