# Supplementary material for: Ten‐year trends of national healthcare costs of asthma, allergic rhinitis, and atopic eczema in 3 million Norwegians
Source: Allergy. 2022 Feb 1;77(5):1614–6. doi: 10.1111/all.15225 (PMC9303559; doi:10.1111/all.15225)
Supplement: Supplementary file 1 — Appendix S1 [file ALL-77-1614-s001.docx]

# Appendix supporting the letter:

**Ten year trends of national healthcare costs of asthma, allergic rhinitis and atopic eczema in 3 million Norwegians**

Christine Louise Parr^1,2^, Wenche Nystad^2^, Øystein Karlstad^2^, Knut Øymar^3,4^, Arnulf Langhammer^5^, Per Nafstad^6^, Torbjørn Wisløff^7,8^

^1^ Norwegian Scientific Committee for Food and Environment, Norwegian Institute of Public Health, Oslo, Norway

^2^ Department of Chronic Diseases and Ageing, Norwegian Institute of Public Health, Oslo, Norway

^3^ Department of Pediatrics, Stavanger University Hospital, Stavanger, Norway

^4^ Department of Clinical Science, University of Bergen, Norway.

^5^ Department of Public Health and Nursing, Norwegian University of Science and Technology, Trondheim, Norway

^6^ Department of Community Medicine and Public Health, University of Oslo, Oslo, Norway

^7^ Health Services Research Unit, Akershus University Hospital, Lørenskog, Norway

^8^ Department of Method Development and Analytics, Norwegian Institute of Public Health, Oslo, Norway

**Detailed methods**

All analyses were based on the Norwegian population aged 0-44 years (around 57% of the total population, all ages). This age cut-off was applied because asthma becomes increasingly difficult to separate from chronic obstructive pulmonary disease (COPD) in older adults.^1^

Cost estimations were performed for three areas within the health care sector: 1) drug expenditures 2) specialist health care services (hospitalizations and outpatient visits) and 3) general practitioner (GP) consultations in primary care. We estimated the direct costs attributed to specific diagnoses, which is the most commonly used method in cost of illness studies.^2,3^ Data on patients with reimbursement codes for asthma, allergic rhinitis, and atopic eczema was extracted from the Norwegian Prescription Database (NorPD), the Norwegian Patient Registry (NPR) the Control and Payment of Health Reimbursement Registry (KUHR). Available years were 2008-2017 for NPR, 2006-2016 for KUHR and 2008-2018 for NorPD, but NorPD data was analysed from 2010 when the introduction of detailed reimbursement codes was completed for all relevant outcomes. We use the same methodology to enable comparisons between diseases and between sectors over time.

Data on hospitalization and outpatient visits were extracted from NPR for diagnoses coded according to the 10th revision of the International statistical classification of diseases and related health problems (ICD-10) representing asthma, including bronchiolitis (bronchiolitis J21, asthma J45 and acute severe asthma J46), allergic rhinitis (J30) and atopic eczema (L20) as primary or secondary diagnosis. Bronchiolitis is common in young children and was included in the asthma diagnosis due to symptom overlap. Costs of hospital admissions and outpatient visits were based on matching ICD-10 codes with average costs per diagnosis related group as presented by the Norwegian Directorate of Health in their yearly report from 2018.^4^ Single patient contacts with more than one code were assigned the highest cost based on the same standards being applied by the Directorate of Health.^4^

Data on GP consultations were extracted from KUHR for all visits with any of the International Classification of Primary Care (ICPC-2) codes for asthma (R96), allergic rhinitis (R97) and atopic eczema (S87). For all visits, reimbursement codes as reported to the government agency HELFO (The Norwegian Health Economics Administration) were basis for cost calculations. According to the Norwegian Medicines Agency, the actual costs of GP visits are on average twice the reimbursement fee.^5^ Given that this principle is to be used in reimbursement applications to the Norwegian Medicines Agency, we applied the same principle when costing GP visits in our analysis.

A list of drugs used for the treatment of each of the conditions asthma, allergic rhinitis, and atopic eczema was developed in collaboration with clinicians. Data on these drugs were extracted from NorPD for diagnoses coded according to ICD-10 or ICPC-2, corresponding to specialist health care or primary care prescriptions, respectively. The same codes were used for asthma (J45/R96), allergic rhinitis (J30/R97) and atopic eczema (L20, S87) as in NPR and KUHR, but ICD-10 codes for acute severe asthma (J46) and bronchiolitis (J21) were only part of the asthma diagnosis in NPR (hospitalizations and outpatient visits). Drugs were classified according to the Anatomical Therapeutic Chemical (ATC) classification system. ATC codes included D02AE01, D07A, D07B, D07X, D11AH01, D11AH02, D11AH04, H02AB, L04AD01, N05BB01, R01AC, R01AD, R03AC02-04, R03AC12-19, R03AK, R03BA, R03DC, R03DX05, R03DX08-10, R06AB02, R06AD01, R06AE07, R06AE09, R06AX13, R06AX22, R06AX26, R06AX27 and V01AA. To ensure that drugs were prescribed specifically for the conditions of interest, we based the analyses on filled prescriptions that have been reimbursed (general reimbursement or individually approved) for asthma, allergic rhinitis or atopic eczema. We included prescriptions if they had both an ATC-code and reimbursement code for the condition. We used the in-house software PREVALENS at the Norwegian Institute of Public Health to extract aggregated data from NorPD. For each disease defined by a list of ATC-codes, the sales prices were extracted, grouped by year of dispensing, age, and sex. For all drugs, the exact sales price was used in the calculation of costs.

We present annual cost estimates as figures showing time trends by health outcome and age group. For each sector, costs are first presented separately by health outcome (asthma, allergic rhinitis and atopic eczema), then the overall costs (summarized for outcomes) are presented as the total, or as the average costs for each age group to adjust for differences in population size. The age groups were defined as infants (age 0-<2 years), preschool children (age 2-<6 years), schoolchildren (age 6-<13 years), adolescents (13-<18 years), and adults (18-44 years) in NorPD, but other age groups were used in NPR and KUHR because data were pre-aggregated. The average cost was calculated by dividing the cost in each age group by the number of age cohorts within the group. All costs were standardized to the year 2018.

**Potential explanations**

In Norway, the declining cost of specialist health care for asthma seen during the past decade, has mainly occurred in children under 5 years of age. During this time, the prevalence of prescriptions for anti-inflammatory treatment reimbursed for asthma has also declined among young children, and remained relatively stable in other age groups. Thus, declining costs seem to have other explanations than more extensive regular anti-inflammatory treatment. Declining costs are consistent with a national policy of substituting inpatient care with less costly outpatient- and day care.^6^ Improved handling of asthma exacerbations in emergency rooms (ERs), including increased use of short courses of oral glucocorticoids could lead to fewer hospital admissions. Environmental exposures could also play a role. Norway implemented smoke-free legislation in 2004, which has been associated with a significant reduction in hospital attendance for asthma exacerbations and lower respiratory tract infections in children.^7^

Reductions in the maximum prices for prescription drugs set by the Norwegian Medicines Agency and increasing market shares of generic pharmaceuticals have probably contributed to lower drug expenditures. The reason for the lower cost of GP consultations for asthma is less clear, but better symptom prevention from better treatment or compliance, could play a role.

As for allergic rhinitis costs, higher levels and longer periods of birch pollen exposure due to climate change could potentially lead to more allergic rhinitis in Northern Europe.^8^ However, other factors such as more active treatment in general, and more use of allergen immunotherapy could also drive the costs of both GP consultations (more frequent visits, in particular for subcutaneous administration) and drugs upward.^9^ For atopic eczema, the sharp increase in prescription cost in 2015 coincides with topical carbamide being added to the list of reimbursable treatments in Norway. Moisturizing cream was previously not subject to reimbursement, which partly explains the increase. But the uptrend in prevalence of reimbursed prescriptions, and cost of GP consultation began prior to 2015, suggesting that atopic eczema could be on the rise, although heightened awareness among parents of young children, and/or more active treatment could also be contributing factors.

**Acknowledgements**

We are thankful to Jan Abel Olsen and Maria C. Magnus for comments on a previous version of this work. We are also thankful to Øystein Aalstad Jonasson for providing data and Kristine Olsen for input regarding NorPD.

The current study was supported by the Norwegian Directorate of Health.

**Table 1 Cost of asthma, allergic rhinitis and atopic eczema by health care sector for (NOK, million) in 2016 compared with 2010 (data available in all registries)**

|  | Cost 2016, NOK mill | | | |  |
| --- | --- | --- | --- | --- | --- |
|  | Asthma | Rhinitis | Eczema | Sum sector | Sum sector  (% of total) |
| Drug expenditures (NorPD) | 207 | 151 | 36 | 394 | 45% |
| Specialist health care* (NPR) | 207 | 14 | 29 | 250 | 28% |
| GP consultations (KUHR) | 124 | 63 | 52 | 239 | 27% |
| Sum disease | 538 | 228 | 117 | 883 |  |
| Sum disease, % of total | 61% | 26% | 13% |  |  |
|  | Cost 2010, NOK mill. | | | |  |
|  | Asthma | Rhinitis | Eczema | Sum sector | Sum sector  (% of total) |
| Drug expenditures (NorPD) | 297 | 101 | 16 | 414 | 40% |
| Specialist health care* (NPR) | 354 | 12 | 25 | 390 | 38% |
| GP consultations (KUHR) | 132 | 48 | 39 | 219 | 21% |
| Sum disease | 782 | 160 | 80 | 1 022 |  |
| Sum disease, % of total | 76 % | 16 % | 8 % |  |  |
|  | Cost difference (2016-2010) NOK mill. | | | |  |
|  | Asthma | Rhinitis | Eczema | Difference sector | Difference sector,% of 2010 sum |
| Drug expenditures (NorPD) | -90 | 51 | 20 | -19 | -5 % |
| Specialist health care* (NPR) | -146 | 2 | 4 | -140 | -36 % |
| GP consultations (KUHR) | -8 | 15 | 13 | 20 | 9 % |
| Difference disease | -244 | 68 | 36 | -139 | -14 % |
| Difference disease ,  % of 2010 sum | -31 % | 43 % | 45 % |  |  |

*Specialist health care includes costs of both hospitalizations and outpatient care

**Appendix Figure 1 (as Figure 2; Time trend in the average annual drug expenditures on asthma, allergic rhinitis, and atopic eczema in the Norwegian population aged 0-44 years, by age group. Norwegian Prescription Database 2008-2018.)**


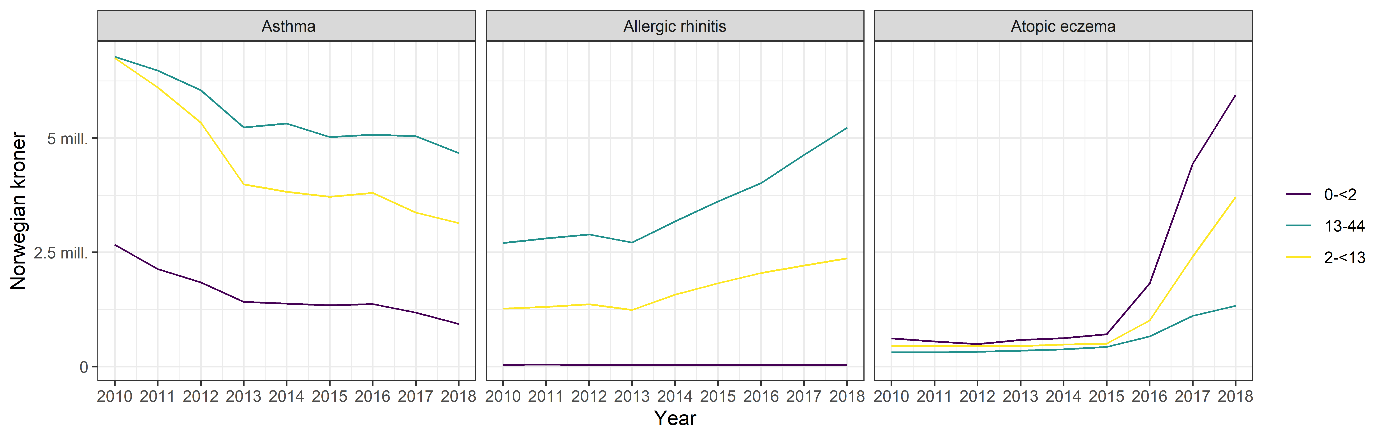


**Appendix Figure 2 Time trend in the average annual costs of hospital and outpatient care for asthma*, allergic rhinitis, and atopic eczema in the Norwegian population aged 0-44 years, by age group. Norwegian Patient Registry (2008-2017).**


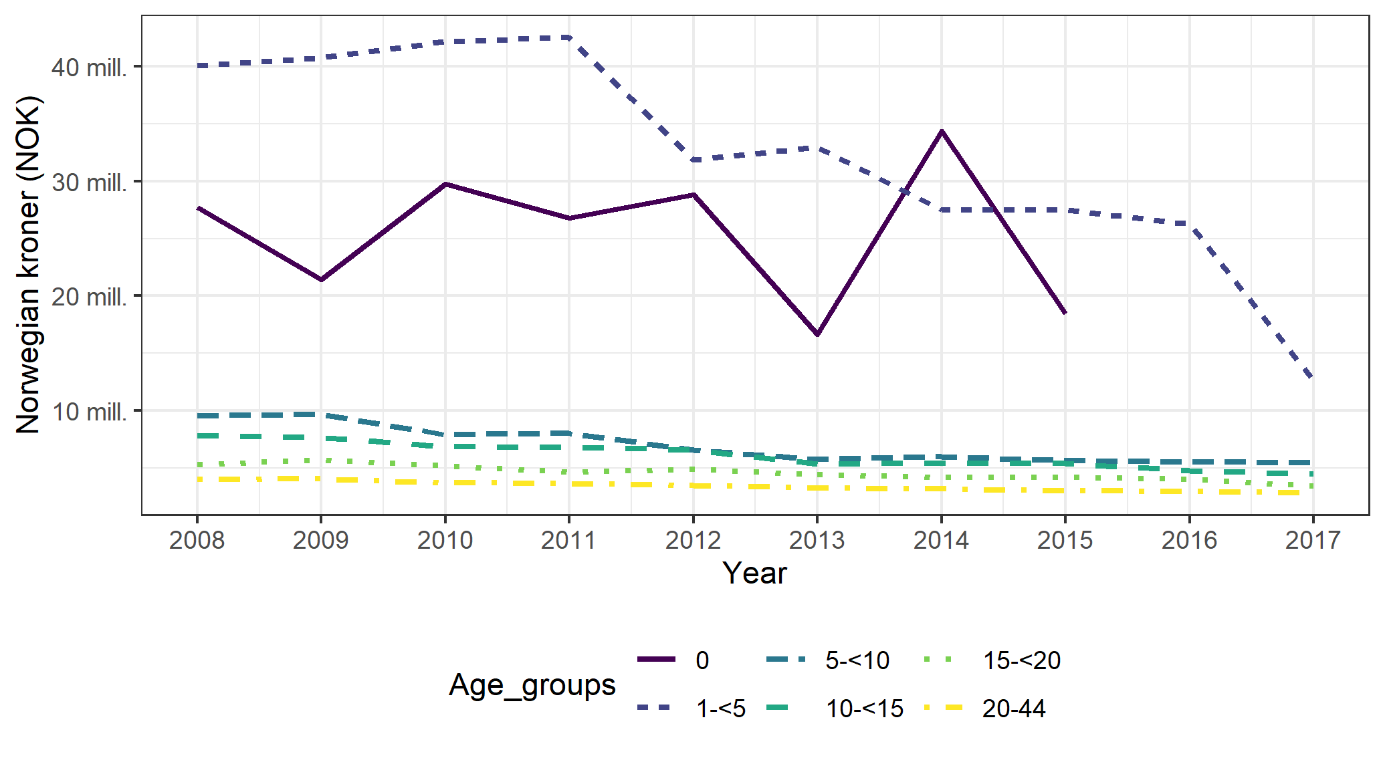


*The asthma diagnosis based on the Norwegian Patient Registry also included bronchiolitis

**Appendix Figure 3 Time trend in the annual costs of GP consultations for asthma, allergic rhinitis, and atopic eczema in the Norwegian population aged 0-44 years, by disease. Control and Payment of Health Reimbursement Registry (2006-2016).**


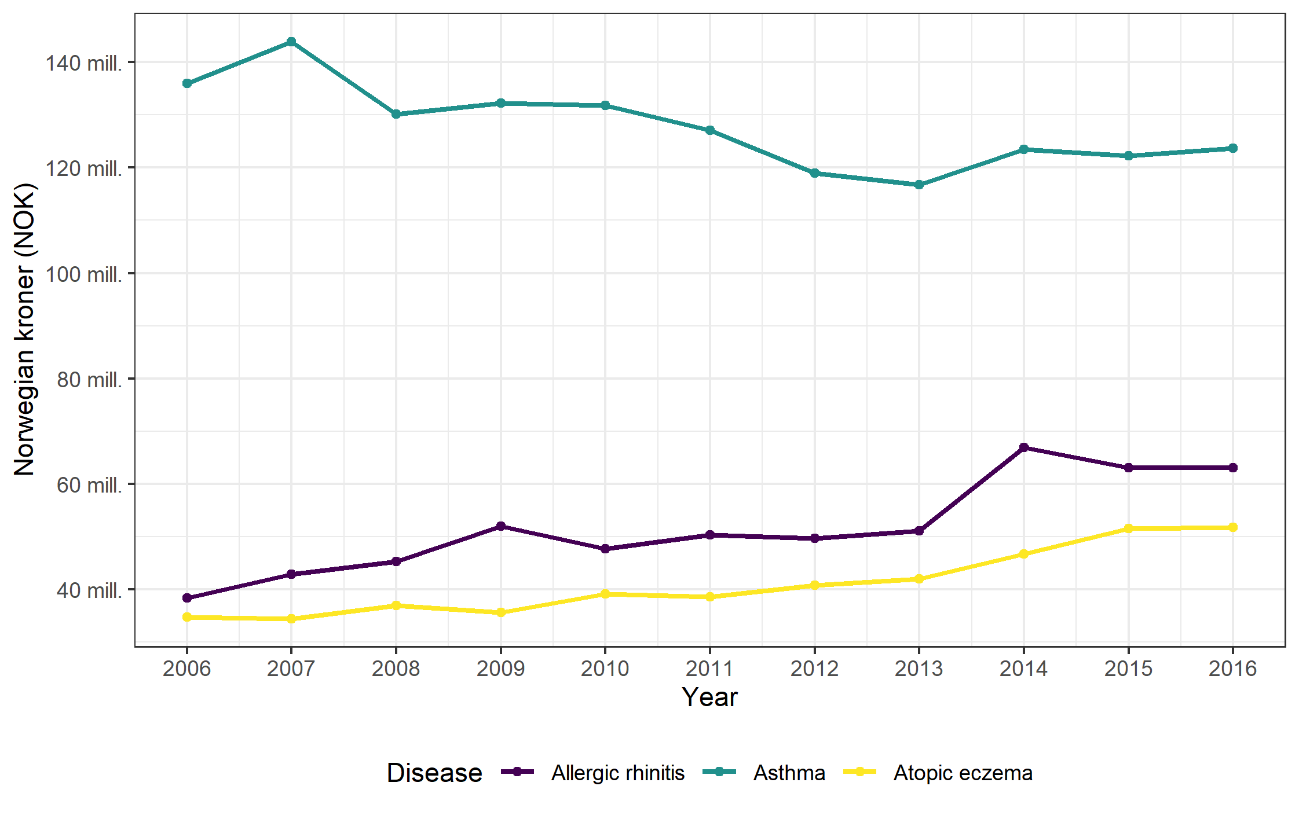


**Appendix Figure 4 Time trend in the average annual costs of GP consultation in primary care for asthma, allergic rhinitis, and atopic eczema in the Norwegian population aged 0-44 years, by age group. Control and Payment of Health Reimbursement Registry (2006-2016)**


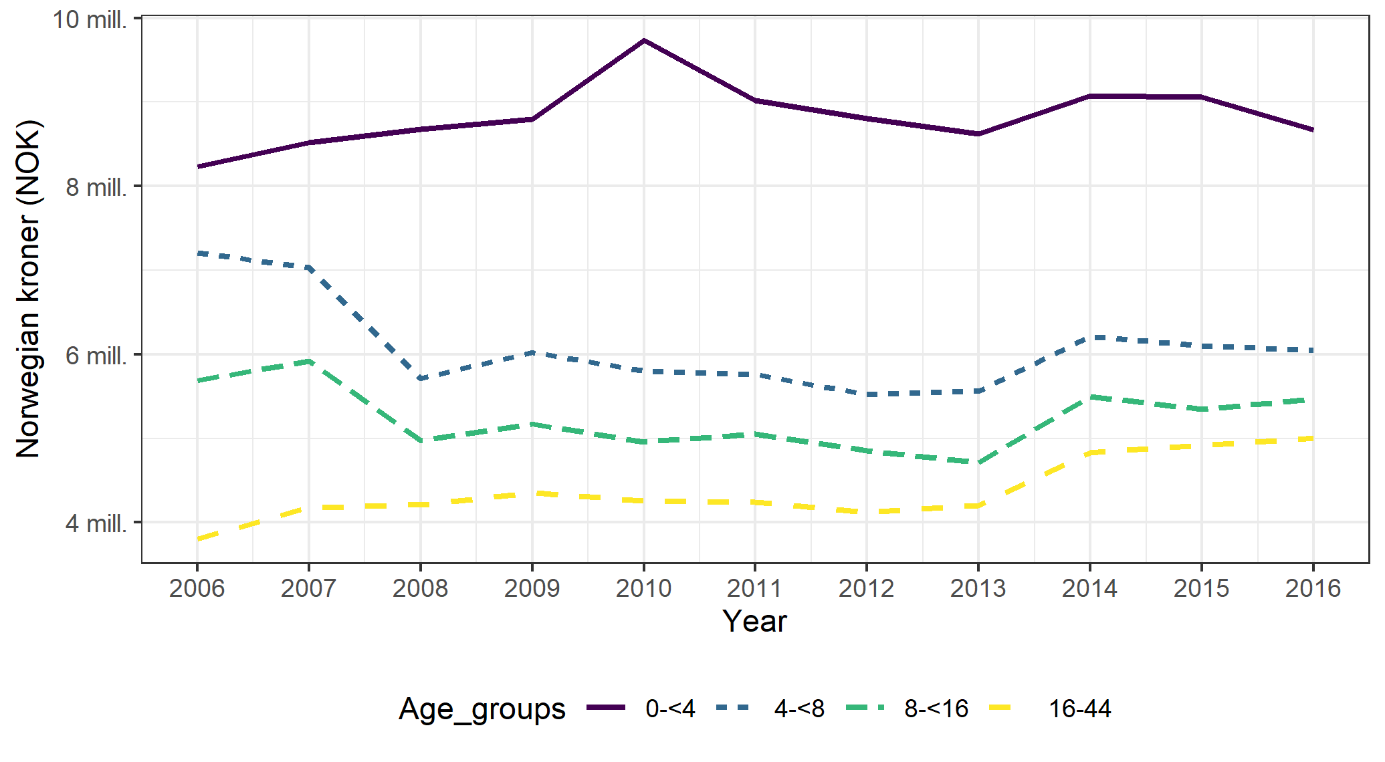


# **References**

1. Burney PG, Luczynska C, Chinn S, Jarvis D. The European Community Respiratory Health Survey. Eur Respir J. 1994 May. Eur Respir J 1994 May;7(5):954-60.

2. Akobundu E, Ju J, Blatt L, Mullins CD. Cost-of-illness studies: a review of current methods. *Pharmacoeconomics* 2006; **24**(9): 869-90.

3. Onukwugha E, McRae J, Kravetz A, Varga S, Khairnar R, Mullins CD. Cost-of-Illness Studies: An Updated Review of Current Methods. *Pharmacoeconomics* 2016; **34**(1): 43-58.

4. Innsatsstyrt finansiering 2018 (in Norwegian). [Stakeholder financing]. Report IS-2689. Oslo, Norway: Norwegian Directorate of Health, 2017.

5. Guidelines for the submission of documentation for single technology assessment (STA) of pharmaceuticals. Oslo; Norway: Norwegian Medicines Agency, 2018.

6. Ringard Å, Sagan A, Sperre Saunes I, Lindahl AK. Norway: health system review. Health Syst Transit 2013; 15(8): 1-162. 26.

7. Faber T, Kumar A, Mackenbach JP, et al. Effect of tobacco control policies on perinatal and child health: a systematic review and meta-analysis. Lancet Public Health 2017; 2(9): e420-e37.

8. Biedermann T, Winther L, Till SJ, Panzner P, Knulst A, Valovirta E. Birch pollen allergy in Europe. Allergy 2019; 74(7): 1237-48.

9. Björstad Å, Cardell LO, Hahn-Pedersen J, Svärd M. A Cost-Minimisation Analysis Comparing Sublingual Immunotherapy to Subcutaneous Immunotherapy for the Treatment of House Dust Mite Allergy in a Swedish Setting. Clin Drug Investig 2017; 37(6): 541-9.
